# Conversational Artificial Intelligence in Medical Education: A Scoping Review

**DOI:** 10.7759/cureus.107391

**Published:** 2026-04-20

**Authors:** Tasniya Aktar, Robert J Farquhar, Chris Jacobs

**Affiliations:** 1 Faculty of Life Sciences and Medicine, King's College London, London, GBR; 2 Department of Psychology, University of Bath, Bath, GBR; 3 Postgraduate Medical Education, Great Western Hospitals NHS Foundation Trust, Swindon, GBR

**Keywords:** artificial intelligence in medicine, chatbots, conversational artificial intelligence, medical education curriculum, medical education technology

## Abstract

Conversational artificial intelligence (AI), encompassing chatbots and large language models (LLMs), is rapidly emerging in the sphere of medical education as a dynamic tool for interactive learning. By generating realistic dialogue, simulating patient encounters, and providing adaptive feedback, these systems create new possibilities for learners to practise communication, clinical reasoning, and decision-making in a flexible and accessible way. Yet, despite increasing enthusiasm, evidence regarding their educational value remains fragmented. This scoping review examines the existing literature on conversational AI in undergraduate medical education, focusing on three key domains: educational utility, technology usability, and fidelity.

A comprehensive search was conducted across PubMed, Scopus, and Web of Science in August 2025. After deduplication, 496 unique studies were screened, and 20 met the inclusion criteria. These studies employed diverse methodologies and evaluation approaches. Methodological rigour was assessed using a validated framework designed for medical education research.

Across the literature, conversational AI demonstrates considerable potential to enhance engagement, support self-directed learning, and expand access to experiential practice. Learners generally view these systems as intuitive and motivating, and many studies suggest benefits for clinical reasoning and communication training. However, limitations in reliability, realism, and technical accuracy persist, and outcome measures remain inconsistent. Few studies assess the impact of response latency or the long-term transfer of skills to clinical settings, and methodological rigour is often modest.

Overall, conversational AI appears to be a promising adjunct to traditional medical teaching rather than a replacement. Its value lies in scalability, interactivity, and adaptability, but effective integration requires thoughtful design, validated evaluation frameworks, and ongoing human oversight. As technology advances, further research should focus on standardising assessment methods, exploring learning outcomes beyond user satisfaction, and addressing fidelity and responsiveness to ensure meaningful, safe, and sustainable implementation within modern medical curricula.

## Introduction and background

Conversational artificial intelligence (AI), including chatbots and large language models (LLMs) such as ChatGPT, is rapidly gaining attention as a potential tool in medical education. LLMs are pre-trained AI systems built to process and understand human language [1]. These systems are capable of generating human-like dialogue, simulating patient encounters, and providing tailored feedback to learners [2]. The result is a novel mode of interaction that can support training in communication skills, knowledge reinforcement, and clinical reasoning [3]. 

The appeal of conversational AI lies in its flexibility and scalability. Virtual patients can be available at any time, allowing students to practise history-taking or clinical reasoning outside of scheduled teaching sessions [4]. Learners can make mistakes in a safe environment, receive immediate feedback, and repeat scenarios as often as needed, features that align closely with principles of deliberate practice and experiential learning [5].

However, enthusiasm alone is insufficient for responsible integration into curricula. Three dimensions are particularly important for educational evaluation. Educational utility examines whether these systems lead to meaningful improvements in learning and assessment outcomes. Technology usability considers whether they are intuitive, reliable, and accessible enough to be adopted by learners and educators, aligning with theoretical frameworks such as Davis’s Technology Acceptance Model [6]. Fidelity assesses the realism of AI interactions relative to human or standardised patients, a critical factor for communication and simulation-based training [7].

Despite a rapidly growing body of literature, the evidence base remains fragmented. Studies differ widely in design, outcomes, and definitions of success. Some report clear benefits, while others note issues such as factual inaccuracies, lack of consistency, or limited generalisability [8]. The aim of this study is to address these uncertainties and conduct a scoping review to map existing research, synthesise findings across educational utility, usability, and fidelity, and identify gaps, with the objective to inform educators on the appropriate use of conversational AI in medical education.

## Review

Methods

The scoping review protocol, including project aims and search methods, was preregistered on the Open Science Framework (project ID: XMN8J) [9]. A comprehensive search was conducted in PubMed, Scopus, and Web of Science on 27/08/25. Search strategies included Boolean terms (Appendix A) covering chatbots, conversational AI, dialogue systems, large language models (e.g., ChatGPT and GPT-4), and virtual patients combined with medical education and outcomes related to educational utility, usability, and fidelity. Two hundred and ninety-two records were yielded by PubMed, 358 by Scopus, and 227 by Web of Science. A total of 877 articles were identified. Three hundred and eighty-one duplicates were detected using Rayyan software (Rayyan Systems Inc., Cambridge, MA), and 238 were removed automatically, with an additional 143 resolved manually. After deduplication, 496 unique records were screened.

**Figure 1:**
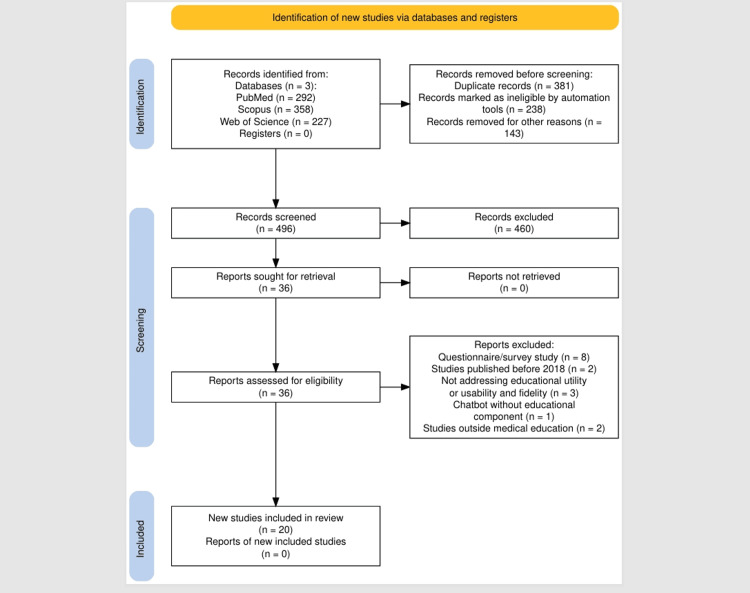
Identification of new studies via databases and registers following PRISMA 2020 guidelines

Two authors independently screened titles and abstracts, with conflicts resolved through consensus. Full-text review was conducted for articles meeting the inclusion criteria. The inclusion criteria and exclusion criteria are displayed in Figure 1.

**Table 1:** Inclusion and exclusion criteria for the scoping review

Inclusion criteria	Exclusion criteria
Participants: medical students actively enrolled in undergraduate medical education	Studies outside medical education (including studies with allied health professions)
Interventions: conversational agents, dialogue systems, large language models (ChatGPT, GPT-4) and AI virtual patients	Chatbots designed exclusively for patient care, symptom checking or therapy without an educational component
Outcomes: educational utility, technology usability or fidelity	Studies not addressing educational utility, usability or fidelity
Study designs: quantitative, qualitative, mixed-methods or pilot studies	Opinion pieces, news articles, blogs, non-peer-reviewed content or reviews
Publication date: 2018 or later	Studies published before 2018
Language: English	Not available in English

To appraise the quality of included studies, we applied the Medical Education Research Study Quality Instrument (MERSQI), a validated tool designed for evaluating methodological rigour in medical education research [10]. The MERSQI assesses domains such as study design, sampling and participant numbers, validity of evaluation instruments, detail of statistical analyses, and appropriateness of conclusions relative to data. Two reviewers independently scored all included studies. Any discrepancies of greater than two points on individual domain scores were discussed until consensus was reached. Inter-rater reliability for the two independent reviewers’ MERSQI scores was assessed using a two-way random effects model, absolute agreement, and single-measures intraclass correlation coefficient (ICC(2,1)).

The study selection process is summarised in the PRISMA diagram (Figure 1) [11].

Results

The inter-rater reliability between the two reviewers’ MERSQI scores was good, with an ICC(2,1) of 0.75, indicating consistent scoring across raters. Included studies with their relevant characteristics and given MERSQI scores are displayed in Table 2. 

**Table 2:** Included studies and their MERSQI scores LLM: large language models; MERSQI: Medical Education Research Study Quality Instrument

Study Title	Author(s)	Country	Journal	Year	Method	Results	AI model	MERSQI Score
Using ChatGPT for medical education: the technical perspective	Chan, K. Y.; Yuen, T. H.; Co, M. [12]	Hong Kong/Singapore	BMC Medical Education	2025	Mixed Methods	Positive outcomes reported	ChatGPT	9.5
Implementation and evaluation of an optimized surgical clerkship teaching model utilizing ChatGPT	Huang, Y.; Xu, B. B.; Wang, X. Y.; Luo, Y. C.; Teng, M. M.; Weng, X. J. [13]	China	BMC Medical Education	2024	Experimental Study	Positive outcomes reported	ChatGPT	12.5
Beyond Traditional Simulation: An Exploratory Study on the Effectiveness and Acceptability of ChatGPT-4o Advanced Voice Mode for Communication Skills Practice Among Medical Students	Mukadam, A.; Suresh, S.; Jacobs, C. [14]	United Kingdom	Cureus	2025	Qualitative Study	Mixed outcomes	ChatGPT	10.5
Termbot: A Chatbot-Based Crossword Game for Gamified Medical Terminology Learning	Hsu, M. H.; Chan, T. M.; Yu, C. S. [15]	Taiwan	International Journal of Environmental Research and Public Health	2023	Experimental Study	Positive outcomes reported	Chatbot (not specified)	11
Integrating large language model-based agents into a virtual patient chatbot for clinical anamnesis training	Laverde, N.; Grévisse, C.; Jaramillo, S.; Manrique, R. [16]	Colombia	Computational and Structural Biotechnology Journal	2025	Experimental Study	Positive outcomes reported	LLM (not specified)	6
Virtual Patient Simulations Using Social Robotics Combined With Large Language Models for Clinical Reasoning Training in Medical Education: Mixed Methods Study	Borg, A., Georg, C, Jobs, B., Huss, V., Waldenlind, K., Ruiz, M., Edelbring, S., Skantze, G., Parodis, I. [17]	Sweden	JMIR Med Educ	2025	Mixed Methods	Mixed outcomes reported	LLM (not specified)	9
A Generative Pretrained Transformer (GPT)-Powered Chatbot as a Simulated Patient to Practice History Taking: Prospective, Mixed Methods Study	Holderried, F., Stegemann-Philipps, C., Herschbach, L., Moldt, J. A., Nevins, A., Griewatz, J., Holderried, M., Herrmann-Werner, A., Festl-Wietek, T., Mahling, M. [18]	Germany	JMIR Medical Education	2024	Mixed Methods	Positive outcomes reported	Chatbot (not specified)	7
Assessing medical students' attitudes, performance, and usage of ChatGPT in Jeddah, Saudi Arabia	Alammari, D.; Alamari, E.; Alamri, R.; Alharbi, R.; Felimban, J.; Aljohani, J. [19]	Saudi Arabia	Frontiers in Artificial Intelligence	2025	Survey/Questionnaire	Positive outcomes reported	ChatGPT	8
Development and evaluation of an emergency department serious game for undergraduate medical students	Aster, A., Hütt, C., Morton, C., Flitton, M., Laupichler, M. C., Raupach, T. [20]	Germany	BMC Medical Education	2024	Experimental Study	Positive outcomes reported	Chatbot (not specified)	13
Impact of providing a customized guideline on virtual medical history taking in two serious games for medical education	Aster, A.; Lotz, A.; Laupichler, M. C.; Raupach, T. [21]	Germany	Med Educ Online	2025	Experimental Study	Positive outcomes reported	Chatbot (not specified)	11
Enhancing Medical Student Engagement Through Cinematic Clinical Narratives: Multimodal Generative AI-Based Mixed Methods Study	Bland, T. [22]	USA	JMIR Med Educ	2025	Survey/Questionnaire	Positive outcomes reported	GPT-4	8
LLM-Based Clinical History Taking System: A Persona-Driven Approach	Choi, D., Jung, Y., Kim, J., Oh, N., Oh, H., Lee, S., Seo, J., Kim, T. [23]	South Korea	Studies in Health Technology and Informatics	2025	Experimental Study	Positive outcomes reported	LLM (not specified)	7
A Language Model–Powered Simulated Patient With Automated Feedback for History Taking: Prospective Study	Holderried, F., Stegemann-Philipps, C., Herrmann-Werner, A., Festl-Wietek, T., Holderried, M., Eickhoff, C., Mahling, M. [24]	Germany	JMIR Medical Education	2024	Experimental Study	Positive outcomes reported	Chatbot (not specified)	8
Incorporating ChatGPT in Medical Informatics Education: Mixed Methods Study on Student Perceptions and Experiential Integration Proposals	Magalhães Araujo, S.; Cruz-Correia, R. [25]	Portugal	JMIR Med Educ	2024	Survey/Questionnaire	Positive outcomes reported	ChatGPT	6
A cross-sectional investigation of ChatGPT-like large language models application among medical students in China a	Pan, G. X.; Ni, J. [26]	China	BMC Medical Education	2024	Survey/Questionnaire	Mixed outcomes	ChatGPT	9
Iteratively refined ChatGPT outperforms clinical mentors in generating high-quality interprofessional education clinical scenarios: a comparative study	Qingquan, T.; Feng, R.; Bin, Z.; Jingyu, Z.; Ganglei, L.; Yanwen, Z.; Zequn, Z.; Qiyuan, W.; Shalong, W. [27]	China	BMC Med Educ	2025	Experimental Study	Positive outcomes reported	ChatGPT	10
The Digital Standardized Patient: An Artificial Intelligence Coach for Cultural Dexterity in Surgical Care	Rao, A. S., Lee, R. S., Bott, E., Jiang, S., Jiao, Q., Dacier, B. M., Haider, A., Farrell, S., Ortega, G., Succi, M. D. [28]	USA	J Am Coll Surg	2025	Experimental Study	Positive outcomes reported	LLM (not specified)	6
Development and Evaluation of an Artificial Intelligence-Powered Surgical Oral Examination Simulator: A Pilot Study	Rao, A. S., Prasad, S., Lee, R. S., Farrell, S., McKinley, S., Succi, M. D [29]	USA	Mayo Clinic Proceedings: Digital Health	2025	Experimental Study	Positive outcomes reported	LLM (not specified)	6.5
Utilizing generative conversational artificial intelligence to create simulated patient encounters: A pilot study for anaesthesia training	Sardesai, N., Russo, P., Martin, J., Sardesai, A. [30]	United Kingdom	Postgraduate Medical Journal	2024	Experimental Study	Mixed outcomes reported	Not specified	6
Application of ChatGPT-based blended medical teaching in clinical education of hepatobiliary surgery	Wu, C. H.; Chen, L. W.; Han, M.; Li, Z.; Yang, N. H.; Yu, C. [31]	China	Medical Teacher	2025	Experimental Study	Positive outcomes reported	ChatGPT	13.5

Study Characteristics

Twenty studies met the inclusion criteria and were included in the final analysis (Figure 1). Most studies were published between 2022 and 2025 and were conducted across diverse regions, including North America (n = 8, 40%), Europe (n = 5, 25%), and Asia (n = 7, 35%). Study designs included randomised controlled trials (n = 6, 30%), quasi-experimental studies (n = 5, 25%), cross-sectional surveys (n = 7, 35%), and mixed-methods evaluations (n = 2, 10%). Sample sizes ranged from 28 to 412 participants, with most focusing on preclinical medical students.

Educational Utility

Most included studies reported enhanced learner engagement and improved knowledge retention through the use of conversational AI tools. Chan et al. [12] reported that over 75% of students agreed that virtual bedside teaching with chatbots improved the effectiveness of their learning and overall experience. LLM-driven virtual patients supported history-taking practice and clinical reasoning exercises. For instance, Huang et al. [13] found that students using a digital patient system performed significantly better in history-taking skills than a control group. In addition, Mukadam et al reported a statistically significant improvement in the perceived usefulness of ChatGPT as a tool for communication skill practice before and after use [14].

Technology Usability 

Usability was generally rated positively, although only three of the studies used quantitative tools to measure this aspect. Hsu et al. [15] reported a mean System Usability Scale (SUS) score of 83.25; whilst Laverde et al. [16] described a mean Chatbot Usability Questionnaire (CUQ) score of 86.25. Both of these results demonstrate satisfaction from learners regarding the usability of the chatbots. Learners reported positive findings for the personality of the chatbot, in particular that the chatbot was realistic and engaging. Additionally, they also found the chatbot easy to navigate and not overly complex.

Comparatively, Aster et al. [2] found that learners were not satisfied with the chatbot, reporting a SUS score of 59.19, below the minimum score of 68 for satisfaction. Learners in the study described that limitations with the user interface affected the usability of the chatbot, in particular, the difficulty in finding elements that they required. Furthermore, at times, incoherent answers prevented the students from taking a history.

*Fidelity* 

Fidelity findings were mixed. While LLM-based virtual patients produced realistic dialogue and were perceived as authentic, some studies reported inconsistent or factually incorrect responses. For example, whilst Aster et al. [2] reported a virtual emergency department experience using conversational AI as having an overall positive evaluation, students found the chatbot sometimes answered incoherently when taking a medical history. Other studies, such as Borg et al. [17], evaluating a social robotic platform enhanced by an LLM, reported an ‘authentic and engaging learning experience’ for medical students using conversational AI.

High-fidelity scenarios were particularly valued in communication skills training but required close supervision by educators. Fidelity may also be limited by response latency, the time taken for the chatbot to respond to the user. Research from Kim et al. [32] shows that consumers typically preferred quick responses from chatbots, although this could be offset with tools such as typing indicators. 

Discussion

This scoping review demonstrates growing evidence that conversational AI can enhance medical education across multiple domains. Most studies reported improvements in learner engagement, perceived knowledge gains, and history-taking performance, consistent with prior reviews of digital simulation tools [12]. Importantly, several studies reported statistically significant improvements in objective outcomes such as examination scores and structured clinical assessments [13], suggesting that conversational AI can contribute to measurable skill development when appropriately implemented.

The findings collectively suggest that conversational AI is well-received by learners, with high usability scores and positive attitudes towards its educational value. These results align with theoretical frameworks such as Davis’s Technology Acceptance Model [6], which emphasises perceived usefulness and ease of use as drivers of adoption. The ability to access virtual patients on demand may be particularly advantageous for early learners who need repeated, low-stakes practice before encountering real patients. 

These findings also align with broader research demonstrating that AI-mediated simulation and feedback can improve diagnostic accuracy, communication skills, and learner confidence across various health professional training contexts, reinforcing the growing consensus that AI-supported experiential practice can complement traditional instruction [3,4]. Moreover, this review supports prior literature indicating that conversational AI can lower barriers to practice, enabling distributed and asynchronous learning opportunities that are difficult to provide using standardised patients alone [24].

However, fidelity remains a key challenge. While some studies reported near-human realism in dialogue, others highlighted factual inaccuracies and conversational breakdowns that could undermine learning if not addressed. These findings echo concerns raised in prior literature on virtual patients and AI tutors, where deficits in realism and consistency have been linked to reduced transferability of skills to real-world encounters [13,7]. These issues reduce authenticity and can affect the quality of learning. Several papers, including Zidoun et al. (2024) [33], also reported the need for human supervision. This supports the view that AI tools should be used as an adjunct, not a replacement for human educators. Future work should focus on improving psychological and functional fidelity, as recommended by Hamstra et al. [7].

Strengths

This study undertook an extensive review of multiple databases, with findings that have implications for future practice. Additionally, a risk bias assessment was conducted to review the included study quality to ensure any conclusions drawn were taken into consideration in relation to the types of studies reviewed. 

Limitations

Most included studies demonstrated methodological limitations when appraised using the MERSQI. Overall, MERSQI scores were low, with the majority of studies falling below 12/18, indicating modest methodological rigour. Many papers relied heavily on self-reported outcomes such as learner satisfaction, perceived usefulness, and attitudes toward conversational AI, typically measured through post-intervention surveys or Likert scales. While these findings provide valuable insight into acceptability and feasibility, they do not directly demonstrate improvements in knowledge, skills, or clinical performance. Only a small subset of studies employed pre- and post-test designs or objective performance measures, such as structured history-taking assessments or examination scores, which are critical for establishing causal impact. The relative scarcity of robust, measurable outcomes highlights the need for future research employing experimental designs and validated assessment tools to more definitively determine the educational value of conversational AI.

Another drawback is the lack of standardisation in outcome measures. Studies used heterogeneous tools and metrics, ranging from ad hoc questionnaires to non-validated scales, making it difficult to compare findings across settings or synthesise results in a meaningful way. This variability reduces confidence in the generalisability of current findings and highlights the need for common evaluation frameworks. Fidelity assessments also lacked consistency: some papers provided only subjective impressions of realism, while others omitted fidelity evaluation altogether. The absence of standardised reporting limits the interpretability of AI’s educational value across contexts.

One issue in the assessment of fidelity is that there seems to be a gap in the evaluation of latency. Latency refers to the time delay between an AI system receiving an input, such as user prompting, and the AI output [34]. In examining included studies, there was little or no mention of explicit measurements of latency as a factor affecting the fidelity of AI. For example, the “Generative GPT-Powered Chatbot as a Simulated Patient for History Taking” [18] provides detailed data on answer plausibility, alignment with script, and usability, but does not mention how long the system takes to respond or whether students perceived any waiting time as problematic. Likewise, the Virtual Patient Simulations Using Social Robotics Combined With Large Language Models study identifies technical/user‐related limitations but does not report metrics or comments about delay or latency in response between student input and AI output. Latency is known from other literature to degrade user experience in interactive systems [35], therefore suggesting this may be an undervalued barrier to usability, realism, and learner satisfaction. Future work should explicitly measure system response times, report participant observations about delays, and test whether latency correlates with reduced learning benefit, lower perceived fidelity, or drop-off in engagement.

Future Directions

Addressing these gaps requires the development and adoption of standardised metrics for evaluating conversational AI in medical education. Future research should draw upon established, validated measures such as the SUS for usability, fidelity frameworks that distinguish between functional, psychological, and interaction fidelity, and educational value scales that capture both objective learning outcomes and motivational impact. Incorporating constructs from Self-Determination Theory [36] could also help capture the role of intrinsic motivation in sustaining learner engagement with conversational AI.

Longitudinal designs are especially needed. Few studies assessed whether knowledge and skills gained with conversational AI persist beyond the immediate training encounter. Future research should therefore include longer follow-up intervals and use objective measures such as communication-focused Objective Structured Clinical Examinations (OSCEs) or observed workplace performance to evaluate skill retention and transfer to clinical practice. Standardisation of outcome measures across trials would not only enable meta-analyses but also facilitate meaningful benchmarking of AI tools against established modalities such as standardised patients.

## Conclusions

Conversational AI holds promise to complement traditional medical education, particularly for communication skills training, history-taking practice, and reinforcement of clinical reasoning. Evidence from this review suggests high learner satisfaction and strong usability in medical education. However, fidelity concerns and the risk of factual inaccuracies highlight the importance of supervised use and iterative refinement. This review highlights that, with careful integration, conversational AI could become an integrated tool for delivering scalable, accessible, and engaging medical education.

## Appendices

Appendix A 

**Table 3:** Search terms

Database	Search Strategy	Date Searched
PubMed	("Chatbots"[Mesh] OR chatbot* OR "conversational AI" OR "conversational agent*" OR "dialogue system*" OR "large language model*" OR LLM* OR "generative AI" OR ChatGPT OR "GPT-4" OR "virtual patient*" OR "intelligent tutoring system*") AND ("Education, Medical"[Mesh] OR "medical education" OR "health professions education" OR "clinical education" OR "undergraduate medical education" OR "graduate medical education" OR "continuing medical education" OR "medical student*" OR resident* OR "patient simulation"[Mesh] OR "simulation training" OR OSCE) AND ("educational utility" OR "learning outcome*" OR "teaching effectiveness" OR "training outcome*" OR usability OR "user experience" OR UX OR "Technology Acceptance Model" OR TAM OR "System Usability Scale" OR SUS OR fidelity OR realism OR authenticity OR "simulation fidelity")	27/08/25
Scopus	TITLE-ABS-KEY ((chatbot* OR "conversational AI" OR "conversational agent*" OR "dialogue system*" OR "large language model*" OR ChatGPT OR "generative AI" OR "virtual patient*") AND ("medical education" OR "health professions education" OR "clinical education" OR "medical student*" OR resident* OR "simulation training" OR OSCE OR "patient simulation") AND ("educational utility" OR "learning outcome*" OR "teaching effectiveness" OR usability OR "user experience" OR "Technology Acceptance Model" OR TAM OR "System Usability Scale" OR SUS OR fidelity OR realism OR authenticity OR "simulation fidelity"))	27/08/25
Web of Science	TS=(chatbot* OR "conversational AI" OR "conversational agent*" OR "dialogue system*" OR "large language model*" OR ChatGPT OR "generative AI" OR "virtual patient*") AND TS=("medical education" OR "health professions education" OR "clinical education" OR "medical student*" OR resident* OR "simulation training" OR OSCE OR "patient simulation") AND TS=("educational utility" OR "learning outcome*" OR "teaching effectiveness" OR usability OR "user experience" OR "Technology Acceptance Model" OR TAM OR "System Usability Scale" OR SUS OR fidelity OR realism OR authenticity OR "simulation fidelity")	27/08/25
